# Ambulatory inguinal hernia repair in Portugal - a multicenter prospective cohort study

**DOI:** 10.1007/s13304-025-02084-6

**Published:** 2025-03-25

**Authors:** PTSurg (Portuguese Surgical Research Collaborative), Alice Pimentel, Teresa Santos, Sofia Dias da Silva, Lúcia Carvalho, Ana Luísa Pinto Frutuoso, Rita Matias, Leonor Matos, Filipe Almeida, Fabiola Amado, Alexandra Ferreira, Isabel Martins, Estanislau Mateia, Vanessa Praxedes, Joana Seabra, Xavier de Sousa, André Silva, Márcia Carvalho, João Mendes, Carlos Macedo Oliveira, Francisco Girão de Caires, Ana Luísa Rodrigues, Regina Silva, Rui Lacerda Cunha, Ana Rita de Sousa Marinho Falcão, Ester Ferreira, Carla Menezes, Inês Neri, Rafael de Castro Nobre, Ana de Clamouse Rebelo, Pedro Santos, David Ferra de Sousa, Ana Andrade, Inês Barros, Sofia Frade, João Gomes, Inês Nunes, Sofia Pina, Nádia Silva, Rui Sousa, Aldara Faria, Ana Gomes, Carlota Ramos, Vanessa Santos, Catarina Antão, Luís Castro, Joana Ferreira, Inês Lima, Filipa Policarpo, Sara Ramtula, Joana Romano, Sara Silveira, Joana Romano, Nuno Rombo, Francisco Baeta, Ana Sofia Boligo, Diogo Cardoso, Vasco S. Cardoso, Claúdia Figueiredo, Isabela Gil, Ana Rita Monte, Joana Romano, Constança M. Azevedo, Rui Cunha, Filipa Dias Mendes, Miguel Semião, Ana Almeida, Maria João Amaral, André Amaro, Andreia Guimarães, Catarina Lopes, Oriana Nogueira, Eva Santos, Marta Rodrigues da Silva, Vítor Devezas, Telma Fonseca, Fábio Gomes, Joana Mafalda Monteiro, António Pereira-Neves, Jorge Nogueiro, Mariana Canelas-Pais, André Pereira, Fernando Resende, Sara Rodrigues, Edgar Amorim, Beatriz Dias, Victor Hugo Baptista, João Melo, Inês Miguel, Juan Rachadell, Antonio Rivero, Liliana Sequeira, Diogo Veiga, Andreia Branco, Inês Costa Carvalho, Barbara Castro, Sofia Fonseca, Raquel Prata Saraiva, Tatiana Queirós, Ana Rita, Alexandra Campos da Silva, Inês Teixeira, Ana Paula Torre, Cátia Cunha, Marisa Peralta Ferreira, Pedro Miranda, Ana M. Cabral, Bárbara Nunes Gama, Catarina dos Santos Rodrigues, Nisalda Carla Melo Rosa, Diogo Galvão, Anaísa Silva, Ana Cláudia Soares, Bárbara Vieira, Ana Couceiro, Marta Ferreira, Narcisa Guimarães, Inês Mónica, Simone Oliveira, Daniela Pais, Hugo Ribeiro, Renato Barradas, Sónia Martins, Miguel Almeida, Ana Faustino, António Freitas, Ana Beatriz Martins, Catarina Moura, Rafaela Parreira, Joana Bolota, Ana Margarida Monteiro Cinza, Sofia Leandro, Rita Lima, Joana Oliveira, Mário Pereira, Miguel Rocha Melo, Cristina Velez, Adalberto Cardoso, Mariana Claro, Ana Cláudia Deus, Andreia Ferreira, Hugo Gameiro, Diogo Marinho, Daniel Costa Santos, Alberto Abreu da Silva, Sara Rodrigues Silva, Diogo Sousa, Ana Lúcia Preto Barreira, Filipe Borges, Pedro Silva Pereira Sousa Botelho, Brigitta Cismasiu, Margarida Silva Ferreira, Susana Henriques, José Guilherme Nobre, Maria Francisca Rodrigues de Areia Brito Da Silva, Ricardo Manuel Branco Souto, César Carvalho, Filipe Guerra, Inês Guerreiro, Paulo Sousa, Filipe André Ramalho de Almeida, David Aparício, Inês Rita Capunge, Rita Marques de Sá Carmarneiro, Jorge Cristo, Marta Fragoso, Joana Frazão, João Guimarães, Ana Rita Martins, Rita Ribeiro Reis Vale Martins, Maria de Jesus Oliveira, João Silva Ribeiro, Paula Soraya de Carvalho e Azevedo Teixeira, Telma Rodrigues Brito, Diana Carina Lima Gomes, Mariana Silva Leite, Carolina Matos, Cristina Ferreira Monteiro, Diogo Abel Vasconcelos Nogueira Pinto, Cristina Silva, Bruno Ribeiro da Silva, Carina Baldino, Ana Guerreiro, Maria Jervis, André Pacheco, Valter Paixão, Vera Pedro, Joana Marantes Pimenta, Filipa Narciso Rocha, Manuela Mega, Rita Monteiro, Joana Peliteiro, Manuela Romano, Alexandra Soares, Mafalda Sampaio-Alves, Natacha Alves, Gabriel Costa, Lígia Freire, José Pedro Gonçalves, Tatiana Marques, Francisco Marrana, Sara Marques, Diogo Melo Pinto, Catarina Quintela, Evgeniya Sitchikhina, Pedro Valente, João Miguel Carvas, Maria Inês Durães, Guida Pires, Carlos Pires, Joana Gomes da Silva, Miguel Brito, Hugo Capote, Cristina Costa, Guilherme Fialho, Tamiris Mogne, Sara Morais, Beatriz Mourato, David Salvador, Coral Aguero, Joaquina Dominguez, Miguel Angel Fernandez, Carlos Figueiredo, Monica Guerrero, Manuel Neuparth, Marta Reia

**Affiliations:** Unidade Local de Saúde de Santa Maria, Lisboa, Portugal

**Keywords:** Hernia, Inguinal, Ambulatory surgical procedures, Postoperative complications, Perioperative care

## Abstract

Ambulatory surgery is the recommended approach for elective inguinal hernia repair for most people. However, the relative use of this procedure in Portugal and its related outcomes are unknown. We aimed to assess complication rates in patients undergoing ambulatory and inpatient surgery. Prospective multicentric cohort study included consecutive patients undergoing elective inguinal hernia repair in mainland Portugal (October–December 2019). The primary outcome was the post-operative complication rate (any Clavien–Dindo grade) among patients undergoing ambulatory and inpatient surgery. A logistic regression analysis was performed to adjust for patient and disease-related co-variables. Eight hundred twenty-eight patients (89.1% of males) were included from thirty-three hospitals, of which seven hundred sixteen (86.4%) had unilateral hernias. Four hundred thirty-three (52.2%) were operated on as day cases and three hundred ninety-five (47.7%) with overnight stays. There were no significant differences in post-operative complication rate between patients undergoing ambulatory and overnight stay surgery, both in unadjusted (9.9% vs. 11.1%; *p* = 0.650) and adjusted (odds ratio: 1.08 [95% CI 0.66–1.76]) analyses. Ambulatory surgery was only performed in half of the patients, although this procedure is not associated with an increased risk of complications. These results should promote the expanded use of ambulatory surgery in patients eligible for elective inguinal hernia repair.

**Supplementary Information:**

The online version contains supplementary material available at 10.1007/s13304-025-02084-6.

## Introduction

Inguinal hernia is a very frequent disease (lifetime risk of 27% in males and 3% in females) [1] whose only definitive treatment is surgical repair. Day-case or ambulatory surgery, pioneered in 1909 by Nicoll [2], was first described for inguinal hernia repair in 1955 [3] and has many potential benefits, such as greater comfort for the patient, less incidence of post-operative delirium and a faster recovery to baseline status [4], making more hospital beds available and contributing toward the reduction of waiting lists, being less labor- and resource-intensive than inpatient treatment [5]. Furthermore, in the post-COVID-19 pandemic era, the need for expanding outpatient care has become even more apparent [6].

Ambulatory surgery is most suitable for low-risk procedures. According to the ACS NSQIP Surgical Risk database, inguinal hernia repair is a relatively safe procedure, with an overall mortality rate of 1.8% and a complication rate of 2% [7], having been initially mainly offered to young and fit patients and now expanding to more complex cases and older patients with higher peri-operative risk [8]. Given the non-inferior clinical outcomes associated with ambulatory surgery reported in the literature [9] and recommendations endorsed in clinical guidelines, its use should be maximized provided patient safety, underscoring the need for careful patient selection and a good liaison between hospital and community services [4].

There is considerable variation in local ambulatory inguinal hernia repair practices worldwide, with rates ranging from as low as 34% of all surgeries in Spain to 87% in the Veneto region in Italy [10]. A substantial proportion of this variation can be attributed to factors other than clinical criteria alone. Currently, in Portugal, there is no database for the registry of hernia-related outcomes.

The Portuguese inguinal hernia cohort (PINE) study is a prospective multi-center cohort study and the first to analyze inguinal hernia repair practices in Portugal comprehensively [11], aiming to characterize its peri-operative management and outcomes better. This cohort was delivered by the Portuguese Surgical Research Collaborative (PTSurg), a collaborative trainee-led research group composed of mainly surgeons in training with a countrywide representation throughout the Portuguese medical landscape. In the presented PINE cohort study, we aimed to estimate the relative use of ambulatory surgery among patients undergoing elective inguinal hernia repair in mainland Portugal and compare complication rates between patients undergoing ambulatory and inpatient surgery.

## Methods

### Study setting and design

The study protocol followed the STROBE guidelines [12] and the STROCCS statement [13] and is fully available at Clinicaltrials.gov (NCT04328597) [14].

**Fig. 1:**
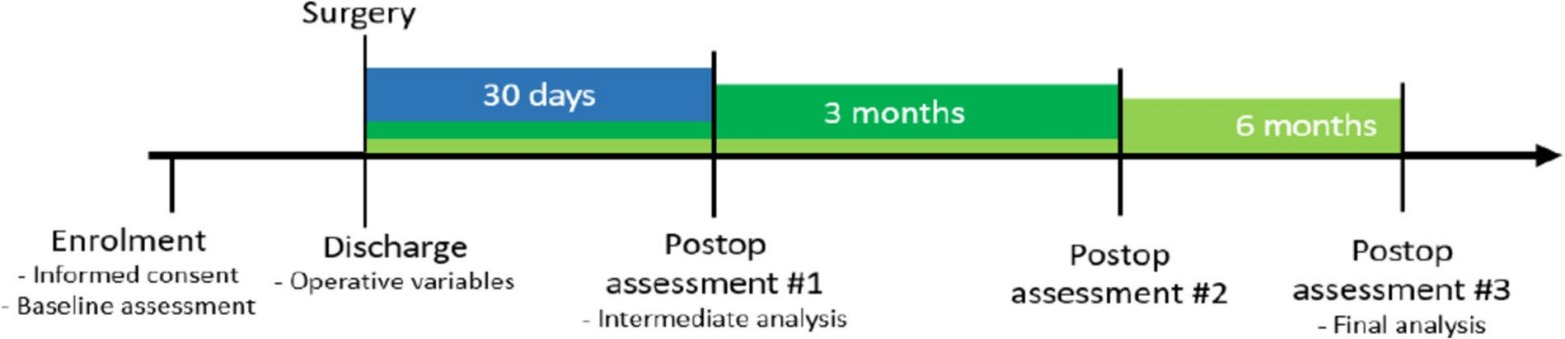
Data collection timeline. With permission from PTSurg, 2020 [14]

In brief, any public mainland Portuguese hospital (33 in total) performing inguinal hernia repair was eligible to participate. Patients were recruited consecutively by the clinical care team at each center during the following 14-day periods (7^th^–18^th^ Oct, 28^th^ Oct–8^th^ Nov, 18^th^ Nov–29^th^ Nov, 29^th^ Nov–13^th^ Dec 2019) and identified through an active search of elective surgery schedules. Participant centers included hospitals of all levels of differentiation and means, ranging from district hospitals to tertiary referral centers. Demographical and surgical data were collected at baseline, and operative data were collected during the immediate pre- and post-operative periods. All patients’ eligibility for ambulatory surgery was assessed and decided at the local investigator’s discretion. Follow-up was obtained 30 days and 3 months after surgery through in-person consultation, telephone or EHR records (Electronic Health Record), as appropriate. A 6 6-month assessment was planned but impossible due to the COVID-19 pandemic. Approval by local ethical committees and written patient consent were obtained [15] (Fig. 1).

All adult patients undergoing elective inguinal repair in Portuguese Hospitals who were willing and able to consent and comply with the follow-up protocol were included. Patients from the original PINE cohort were excluded if they had an inpatient stay greater than one night, as these patients were judged not to have comparable comorbidities to patients undergoing ambulatory care. Emergent repairs were likewise excluded.

### Outcomes and data analysis

The primary outcome was the incidence of post-operative complications defined as any Clavien–Dindo grade I to V, where a higher grade indicates greater severity. [16]. The Clavien–Dindo Classification assigns a grade based on the therapy required to treat a given post-operative complication, enabling one to consistently, reproducibly, and objectively rate post-operative complications. Comprises five grades from I to V, with a greater score indicating complications of greater severity [16].

An initial descriptive analysis of patient and disease-related characteristics was performed, followed by a Chi-square analysis to compare unadjusted outcome rates between patients undergoing ambulatory care and overnight stay. A multivariable logistic regression adjusting for potential patient and surgical characteristics was performed to explore an independent association between peri-operative mode of care (independent variable) and post-operative complications rate (dependent variable). These variables were chosen because they were judged to be sources of confounding between the two groups. The model specification was performed using RStudio *glm* and *finalfit* packages.

*Sensitivity analysis*: patients who were operated in ambulatory care and with overnight stay were expected to have different characteristics and baseline risk of complications, some of them not captured in this study (e.g., use of anticoagulants, lack of social support required for ambulatory surgery). A subgroup analysis included patients judged a priori as low-clinical-risk patients. This was performed to reduce heterogeneity between groups and to highlight a patient population for whom the benefits of promoting ambulatory surgery would be most actionable and had a clear clinical indication for this peri-operative management strategy. Patients with low clinical risk were defined as having an age not greater than 80 years old and an ASA Physical Status less than III.

Data were anonymized, collected, and stored using a secure server using the Research Electronic Data Capture (REDCap) software. Statistical analysis was performed with RStudio 4.4.3 and Rstudio Online. Missing data were collected with the RedCap software and is reported for all tables and figures (including Supplementary Fig. 1). They were excluded from the primary analysis or replaced by the mean/median when applicable.

## Results

### Included and excluded patients

**Fig. 2:**
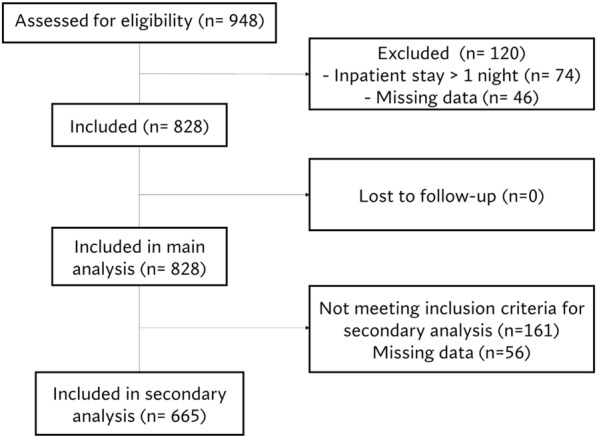
Flow diagram

**Fig. 3:**
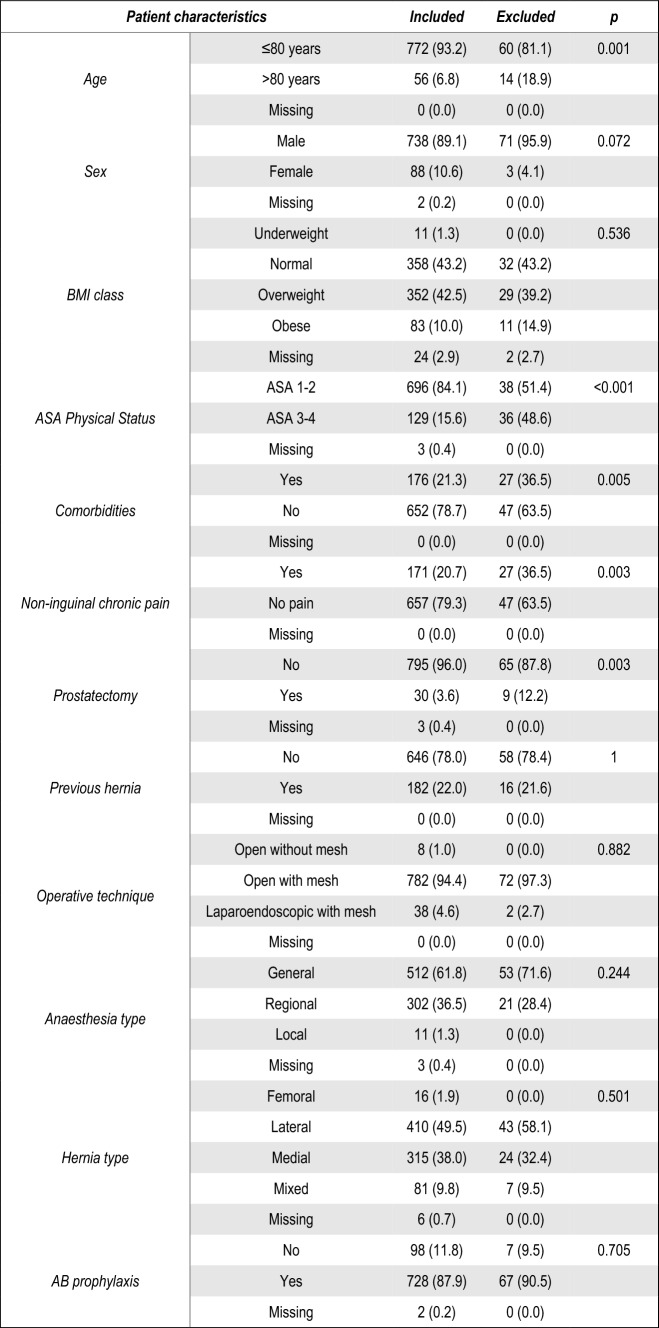
Baseline characteristics of excluded patients

Nine hundred forty-eight patients were included in the PINE study; eight hundred twenty-eight patients (87.3%) were included in the primary analysis, 4.8% had missing data for the primary outcome, and 7.8% were excluded. The excluded patients had a significantly greater number of patients older than 80 years old (6.8% vs 18.9%, *p* < 0.001), a greater ASA Physical Status score (15.6% vs 48.6%, *p* < 0.001), had greater non-inguinal chronic pain (20.7% vs 36.5%, *p* = 0.003) and previous prostatectomy rates (3.6% vs 12.2%, *p* = 0.003) (Figs. 2 and 3).

### Patient characteristics

Eight hundred twenty-eight (87.3%) patients were included in the primary analysis from thirty-three different hospitals. The median age was 61 years, and 89.1% of patients were male. Median BMI was 25.5 kg/m^2^, and 67.9% of patients had ASA 2 Physical Status. Hernia was most frequently unilateral [716 (86.4%) vs 94(11.3%) bilateral] and inguinal [725 (87.5%) inguinal vs 19 (1.9%) femoral]. Of these, 410 (49.5%) were lateral, and 315 (38.0%) were medial inguinal hernias. The most common surgical technique was open mesh repair [782 (94.4%)], the vast majority of which were Lichtenstein [413 (49.8%)] and plug-and-patch repairs [299 (36.1%)]. Overall, 87.9% of patients received AB prophylaxis.

### Ambulatory vs overnight surgery

**Fig. 4:**
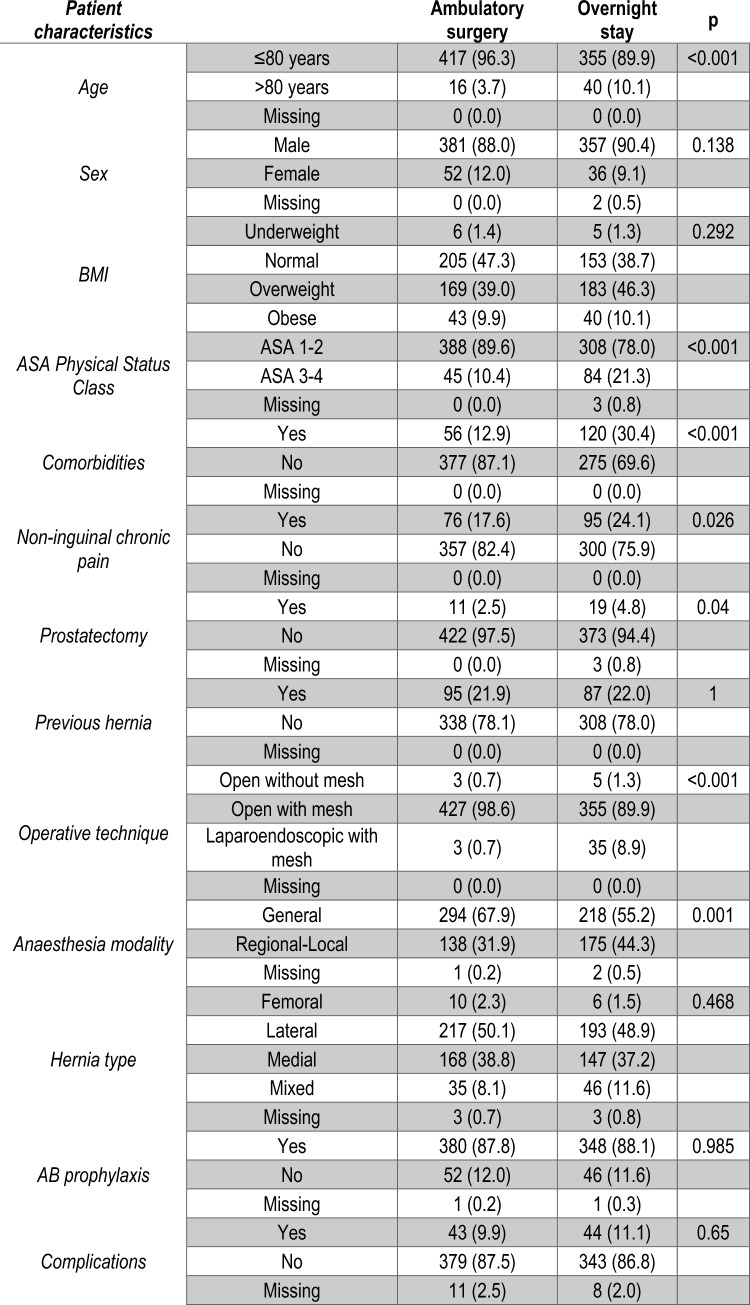
Baseline patient characteristics and rate of post-operative complications by mode of care

Four hundred thirty-three (52.3%) of patients underwent ambulatory and three hundred ninety-five (47.7%) overnight stay. Patients undergoing ambulatory surgery were significantly younger (3.7% aged > 80 vs 10.1%, *p* < 0.001), had lower ASA Physical Status (10.4% with ASA3/4 vs 21.3%, *p* < 0.001), comorbidities (12.9% vs 30.4%, *p* < 0.001), non-inguinal chronic pain rates (17.6% vs 24.1%, *p* = 0.026), and prostatectomy rates (2.5% vs 4.8%, *p* = 0.040), and had higher rates of open mesh repair (98.6% vs 89.9%, *p* < 0.001) and general anesthesia utilization (67.9% vs 55.2%, *p* = 0.001) than those undergoing overnight surgery (Fig. 4).

### Postoperative complications

The complication rate was 10.5% (87 cases for any CD grade). Unadjusted rates of post-operative complications were similar in patients undergoing ambulatory vs. overnight stays (9.9% vs. 11.1%, *p* = 0.650) (Fig. 4).

The differences in post-operative complications remained non-significant in the adjusted logistic analysis, adjusting for age, sex, BMI, previous prostatectomy or inguinal hernia repair, hernia type, operative technique and type of anesthesia, [OR 1.08 (0.66–1.76)] (Supplementary Fig. 2).

### Sensitivity analysis

Six hundred sixty-five patients were included in the sensitivity analysis (low clinical risk). Two hundred forty-two patients were excluded, seventy-four of which were treated as inpatients (7.8%), one hundred sixty-nine had ASA 3 or 4 physical status (17.8%), and seventy-three were older than 80 years old (7.7%). Furthermore, 46 patients had missing data for a mode of care (4.8%). Three hundred seventy-seven (56.7%) patients underwent ambulatory surgery and 288 (43.3%) overnight stay.

The median age was 58, and 88.8% (591/665) of patients were male. The median BMI was 25.4 kg/m2, and 80% (532/665) of patients had ASA 2 Physical Status. Five hundred ninety-three were (89.1%) inguinal hernias [339 (50.9%) lateral and 254 (38.1%) medial)]. Antibiotic prophylaxis was employed in 585 patients (88.0%), and open mesh repair was employed in 627 patients (94.2%), of which 334 (50.2%) were Lichtenstein and 237 (35.6%) were plug-and-patch repairs.

**Fig. 5:**
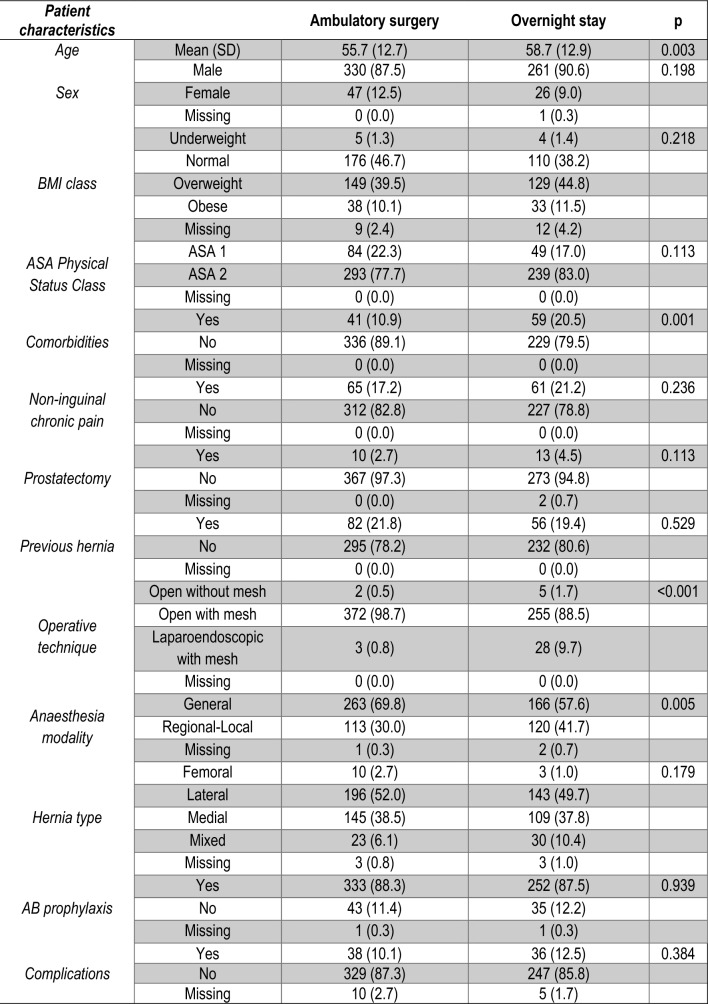
Baseline patient characteristics and rate of post-operative complications by mode of care in selected low-risk patients

In the sensitivity analysis, patients undergoing ambulatory and overnight surgery were significantly different regarding age (*p* = 0.034), operative technique (*p* < 0.001), and anesthesia modality (*p* = 0.002) (Fig. 5).

The post-operative complication rate (74 cases (11.1%)) was not significantly different between the ambulatory surgery and overnight stay groups (10.1% vs. 12.5%, *p* = 0.384) (Fig. 5).

In the adjusted logistic analysis, the differences in complication rates remained non-significant [OR 1.11 (0.65–1.89)] (Supplementary Fig. 3).

## Discussion

This is the first multicentric study in Portugal to analyze inguinal hernia ambulatory surgery coverage and outcomes, including patients from most Portuguese public hospitals. The rate of patients undergoing ambulatory surgery in Portugal is low (52.3%). Although the extant literature recommends coverage of at least 70–80%, approximately half of the total PINE study population received ambulatory surgery, figures comparable to that reported in other European countries [10].

The patient population in this study is representative of current clinical practice, with a median age of 61, meaning most of the patients were of working age. There was a non-negligible proportion (21.2%) of patients with relevant comorbidities (e.g., cardiac, pulmonary, and endocrine). Notably, there was a relatively high rate of open mesh repair (> 90%) and antibiotic prophylaxis (> 85%), significant findings that are also highly dependent on local practice and resources.

Randomized-controlled trials (RCTs) in ambulatory care have previously supported its safety, analyzing a wide range of procedures, though few have explicitly focused specifically on inguinal hernia outcomes [9, 17]. According to Danish databases, the hospital re-admission rate of ambulatory inguinal hernia repair is between 0.8% [18] and 1.1% [19]. The 7-day mortality rate is estimated to be 37 per 100,000 cases. In the present study, there was one death from unknown causes, one case of testicular ischemia and one case of severe hematoma, none of which occurred in the ambulatory care group. In keeping with previous reports, there are no reports of death or severe complications directly related to ambulatory surgery [8].

In this study, we aimed further to probe the safety of ambulatory surgery in inguinal hernia repair. Patients undergoing ambulatory surgery were generally younger and had fewer comorbidities than those undergoing overnight stay. Complication rates of patients undergoing ambulatory surgery were similar to those undergoing overnight stays, even after adjusting for the aforementioned relevant confounding factors (OR 1.08 (0.66–1.76). Notably, in this study, the rate of post-operative complications was comparable to that reported in the literature (9.9%). Still, the incidence of surgical site infections was relatively low (3.7%), possibly contributing to a smaller effect size.

The results remained consistent in the sensitivity analysis of patients with low clinical risk, showing an odds ratio of 1.11 (0.65–1.89). This analysis highlighted a specific group of patients—namely, younger and less frail—who are likely to be good candidates for ambulatory surgery and may require more clinically actionable decisions. These results highlight that although there were significant differences between groups, ambulatory surgery might be a safe but underused alternative. It can optimize patient comfort and contribute to a faster return to baseline function of a patient population largely still in the workforce. Ambulatory inguinal hernia repair diminishes costs by about 25–68%, which could be further reinvested into driving surgical innovation. It also improves patient satisfaction and empowerment, which is increasingly critical in the Patient Reported Outcome Measures (PROMs) era [20].

Future policies should focus on expanding the capacity to provide ambulatory surgery, as it is cheaper for the healthcare system, more comfortable for patients, and has no evidence of a higher complication rate. It should be embraced as the standard of care and as an opt-out option rather than an opt-in alternative. Barriers to the implementation of ambulatory surgery are numerous. Some of the factors identified in this study include socioeconomic factors (no home support or ride home), geographical considerations (living on upper floors of a high building without an elevator), surgeries performed late in the afternoon, meaning the patient recovers from anesthesia too late to be discharged, inadequate post-operative voiding, nausea and pain control and factors relating to anesthetic management (local vs regional vs general anesthesia and pharmacokinetics of different anesthetic drugs). Even among patients judged eligible to undergo ambulatory surgery, many are ultimately treated as inpatients. As can be gleaned by the former discussion, many of these factors can be quickly addressed with careful pre-operative planning with a multidisciplinary focus, with heavy involvement of the social sector and primary care physicians (PCPs), possibly translating in significant healthcare gains for patients, their families, and the healthcare system as a whole, our study contributing to bringing some much-needed attention to this critical problem. Furthermore, the impact of these factors on ambulatory surgery utilization has yet to be adequately studied. It remains an essential area for further study as we strive to bring ambulatory surgery to everyone eligible.

This study represents an important benchmark for further efforts on ambulatory surgery expansion, especially considering Portugal has no national inguinal hernia registry. Recruiting patients from almost all Portuguese public hospitals represents a comprehensive picture of current clinical practice. Although the study was performed in 2019, it remains relevant because it sheds light on essential gaps in clinical practice and highlights an actionable low-clinical-risk patient population that may be underserved in ambulatory surgery.

However, this study presents some limitations. As the PINE Study was not initially powered for this analysis and had an observational study design, we cannot exclude sampling bias. Data on some relevant factors influencing the decision to perform ambulatory surgery were not collected, such as the presence of renal failure, endocrine and metabolic disease, and coagulation disorders. This hindered the analysis of the confounding effect on peri-operative management and complication rate, especially thromboembolism and severe bleeding were among the most common complications reported. Frailty was not assessed in this study, although it has previously been identified as an independent prognostic marker associated with a greater risk of re-admission after ambulatory inguinal hernia repair. In this multi-center study, there was no standardized pre-operative assessment algorithm with the decision to undergo ambulatory surgery dependent on local hospital practices and physician judgement. A protocol for patients’ anesthetic evaluation would be highly valuable in reducing discrepancies in ambulatory surgery inclusion criteria and maximizing adherence to clinical practice guidelines. The Fleisher index [21] predicts which patients are at increased risk of re-admission immediately after ambulatory surgery. However, its inclusion of variables, such as operative time, precludes it from being used as a pre-operative tool to stratify patients and manage care plans efficiently. Highlighting the potential clinical pathway for selecting patients who benefit the most from ambulatory surgery could make a stronger case for its adoption.

## Conclusion

There were no differences in overall post-operative complications between patients undergoing ambulatory surgery and overnight stays after elective inguinal hernia repair, including after adjustment for confounding factors. National and international decision-makers should invest in increasing ambulatory surgery capacity for eligible patients and procedures, particularly as we envision reorganizing our services in the post-COVID era.

## Electronic supplementary material

Below is the link to the electronic supplementary material.

Supplementary file1 (DOCX 97 KB)

## Appendix 1: Collaborative authorship—full authorship

**Writing group**

Andre COUTO DIAS^1^, Joana F.F. SIMÕES^2^, António SAMPAIO SOARES^3^, Costa J^4^ on behalf of PTSurg

1. Unidade Local de Saúde de Santa Maria, Lisboa, Portugal – Corresponding author (andrecoutodias7@gmail.com).

2. Hospital Garcia de Orta. Unidade Local de Saúde de Almada-Seixal. Almada. Portugal.

3. Hospital Professor Doutor Fernando Fonseca. Unidade Local de Saúde de Amadora/Sintra. Amadora. Portugal

4. Laboratory of Clinical Pharmacology and Therapeutics, Faculty of Medicine, University of Lisbon, 1649-028 Lisbon, Portugal; Centre for Evidence-Based Medicine, Faculty of Medicine, University of Lisbon, 1649-028 Lisbon, Portugal; Instituto de Medicina Molecular, Lisbon, Portugal.

PT Surg group members:

Rita Lages, Alice Pimentel, Teresa Santos, Sofia Dias da Silva, Lúcia Carvalho, Ana Luísa Pinto Frutuoso, Rita Matias, Leonor Matos, Filipe Almeida, Fabiola Amado, Alexandra Ferreira, Isabel Martins, Estanislau Mateia, Vanessa Praxedes, Joana Seabra, Xavier de Sousa, André Silva, Márcia Carvalho, João Mendes, Carlos Macedo Oliveira, Francisco Girão de Caires, Ana Luísa Rodrigues, Regina Silva, Rui Lacerda Cunha, Ana Rita de Sousa Marinho Falcão, Ester Ferreira, Carla Menezes, Inês Neri, Rafael de Castro Nobre, Ana de Clamouse Rebelo, Pedro Santos, David Ferra de Sousa, Ana Andrade, Inês Barros, Sofia Frade, João Gomes, Inês Nunes, Sofia Pina, Nádia Silva, Rui Sousa, Aldara Faria, Ana Gomes, Carlota Ramos, Vanessa Santos, Catarina Antão, Luís Castro, Joana Ferreira, Inês Lima, Filipa Policarpo, Sara Ramtula, Joana Romano, Sara Silveira, Joana Romano, Nuno Rombo, Francisco Baeta, Ana Sofia Boligo, Diogo Cardoso, Vasco S. Cardoso, Claúdia Figueiredo, Isabela Gil, Ana Rita Monte, Joana Romano, Constança M. Azevedo, Rui Cunha, Filipa Dias Mendes, Miguel Semião, Ana Almeida, Maria João Amaral, André Amaro, Andreia Guimarães, Catarina Lopes, Oriana Nogueira, Eva Santos, Marta Rodrigues da Silva, Vítor Devezas, Telma Fonseca, Fábio Gomes, Joana Mafalda Monteiro, António Pereira-Neves, Jorge Nogueiro, Mariana Canelas-Pais, André Pereira, Fernando Resende, Sara Rodrigues, Edgar Amorim, Beatriz Dias, Victor Hugo Baptista, João Melo, Inês Miguel, Juan Rachadell, Antonio Rivero, Liliana Sequeira, Diogo Veiga, Andreia Branco, Inês Costa Carvalho, Barbara Castro, Sofia Fonseca, Raquel Prata Saraiva, Tatiana Queirós, Ana Rita, Alexandra Campos da Silva, Inês Teixeira, Ana Paula Torre, Cátia Cunha, Marisa Peralta Ferreira, Pedro Miranda, Ana M. Cabral, Bárbara Nunes Gama, Catarina dos Santos Rodrigues, Nisalda Carla Melo Rosa, Diogo Galvão, Anaísa Silva, Ana Cláudia Soares, Bárbara Vieira, Ana Couceiro, Marta Ferreira, Narcisa Guimarães, Inês Mónica, Simone Oliveira, Daniela Pais, Hugo Ribeiro, Renato Barradas, Sónia Martins, Miguel Almeida, Ana Faustino, António Freitas, Ana Beatriz Martins, Catarina Moura, Rafaela Parreira, Joana Bolota, Ana Margarida Monteiro Cinza, Sofia Leandro, Rita Lima, Joana Oliveira, Mário Pereira, Miguel Rocha Melo, Cristina Velez, Adalberto Cardoso, Mariana Claro, Ana Cláudia Deus, Andreia Ferreira, Hugo Gameiro, Diogo Marinho, Daniel Costa Santos, Alberto Abreu da Silva, Sara Rodrigues Silva, Diogo Sousa, Ana Lúcia Preto Barreira, Filipe Borges, Pedro Silva Pereira Sousa Botelho, Brigitta Cismasiu, Margarida Silva Ferreira, Susana Henriques, José Guilherme Nobre, Maria Francisca Rodrigues de Areia Brito Da Silva, Ricardo Manuel Branco Souto, César Carvalho, Filipe Guerra, Inês Guerreiro, Paulo Sousa, Filipe André Ramalho de Almeida, David Aparício, Inês Rita Capunge, Rita Marques de Sá Carmarneiro, Jorge Cristo, Marta Fragoso, Joana Frazão, João Guimarães, Ana Rita Martins, Rita Ribeiro Reis Vale Martins, Maria de Jesus Oliveira, João Silva Ribeiro, Paula Soraya de Carvalho e Azevedo Teixeira, Telma Rodrigues Brito, Diana Carina Lima Gomes, Mariana Silva Leite, Carolina Matos, Cristina Ferreira Monteiro, Diogo Abel Vasconcelos Nogueira Pinto, Cristina Silva, Bruno Ribeiro da Silva, Carina Baldino, Ana Guerreiro, Maria Jervis, André Pacheco, Valter Paixão, Vera Pedro, Joana Marantes Pimenta, Filipa Narciso Rocha, Manuela Mega, Rita Monteiro, Joana Peliteiro, Manuela Romano, Alexandra Soares, Mafalda Sampaio-Alves, Natacha Alves, Gabriel Costa, Lígia Freire, José Pedro Gonçalves, Tatiana Marques, Francisco Marrana, Sara Marques, Diogo Melo Pinto, Catarina Quintela, Evgeniya Sitchikhina, Pedro Valente, João Miguel Carvas, Maria Inês Durães, Guida Pires, Carlos Pires, Joana Gomes da Silva, Miguel Brito, Hugo Capote, Cristina Costa, Guilherme Fialho, Tamiris Mogne, Sara Morais, Beatriz Mourato, David Salvador, Coral Aguero, Joaquina Dominguez, Miguel Angel Fernandez, Carlos Figueiredo, Monica Guerrero, Manuel Neuparth, Marta Reia.

## Declarations

### Clinical trial number

NCT04328597.
