# Supplementary material for: Ambulatory inguinal hernia repair in Portugal - a multicenter prospective cohort study
Source: Updates Surg. 2025 Mar 25;77(8):2591–600. doi: 10.1007/s13304-025-02084-6 (PMC12630251; doi:10.1007/s13304-025-02084-6)
Supplement: Supplementary file 1 — Supplementary file1 (DOCX 97 KB) [file 13304_2025_2084_MOESM1_ESM.docx]

**Supplementary data**

| **Patient characteristics** |  | **Not missing** | **Missing** | **p** |
| --- | --- | --- | --- | --- |
| Age (years) | 18-40 years | 74 (96.1) | 3 (3.9) | 0.823 |
|  | 41-60 years | 354 (94.7) | 20 (5.3) |  |
|  | 61-80 years | 404 (96.0) | 17 (4.0) |  |
|  | >80 years | 70 (95.9) | 3 (4.1) |  |
| Sex | Male | 809 (95.4) | 39 (4.6) | 1 |
|  | Female | 91 (95.8) | 4 (4.2) |  |
| BMI class | Underweight | 11 (91.7) | 1 (8.3) | 0.333 |
|  | Normal | 390 (95.8) | 17 (4.2) |  |
|  | Overweight | 381 (95.0) | 20 (5.0) |  |
|  | Obese | 94 (98.9) | 1 (1.1) |  |
| ASA Physical Status | ASA 1 | 135 (98.5) | 2 (1.5) | 0.126 |
|  | ASA 2 | 599 (94.8) | 33 (5.2) |  |
|  | ASA 3 | 160 (97.6) | 4 (2.4) |  |
|  | ASA 4 | 5 (100.0) | 0 (0.0) |  |
| Comorbidities | Hepatic | 4 (100.0) | 0 (0.0) | 0.954 |
|  | Pulmonary | 102 (96.2) | 4 (3.8) |  |
|  | Cardiac | 83 (96.5) | 3 (3.5) |  |
|  | Collagen Disorder | 3 (100.0) | 0 (0.0) |  |
|  | Immunosuppression | 11 (100.0) | 0 (0.0) |  |
|  | No | 699 (95.5) | 33 (4.5) |  |
| Non-inguinal chronic pain | Yes | 198 (97.5) | 5 (2.5) | 0.247 |
|  | No pain | 704 (95.4) | 34 (4.6) |  |
| Prostatectomy | No | 860 (96.0) | 36 (4.0) | 0.551 |
|  | Yes | 39 (92.9) | 3 (7.1) |  |
| Previous hernia | No | 704 (95.9) | 30 (4.1) | 1 |
|  | Yes | 198 (95.7) | 9 (4.3) |  |
| Operative technique | Open without mesh | 8 (100.0) | 0 (0.0) | 0.826 |
|  | Open with mesh | 854 (95.7) | 38 (4.3) |  |
|  | Laparo-endoscopic with mesh | 40 (95.2) | 2 (4.8) |  |
| Anaesthesia type | General | 565 (95.9) | 24 (4.1) | 0.7 |
|  | Regional | 323 (95.3) | 16 (4.7) |  |
|  | Local | 11 (100.0) | 0 (0.0) |  |
| Hernia type | Femoral | 16 (100.0) | 0 (0.0) | 0.154 |
|  | Lateral | 453 (96.4) | 17 (3.6) |  |
|  | Medial | 339 (96.0) | 14 (4.0) |  |
|  | Mixed | 88 (91.7) | 8 (8.3) |  |
| AB prophylaxis | No | 105 (95.5) | 5 (4.5) | 1 |
|  | Yes | 795 (95.9) | 34 (4.1) |  |

**Supplementary Figure 1 - Missing data analysis on mode of care**

| **Patient characteristics** |  | **No Complications** | **Complications** | **Multivariate OR** |
| --- | --- | --- | --- | --- |
| Setting | Day-case | 379 (89.8) | 43 (10.2) | - |
|  | Overnight stay | 343 (88.6) | 44 (11.4) | 1.08 (0.66-1.76, p=0.769) |
| Age (years) | 18-40 years | 65 (92.9) | 5 (7.1) | - |
|  | 41-60 years | 300 (89.8) | 34 (10.2) | 1.37 (0.55-4.16, p=0.539) |
|  | 61-80 years | 304 (86.6) | 47 (13.4) | 1.71 (0.68-5.25, p=0.295) |
|  | >80 years | 53 (98.1) | 1 (1.9) | 0.24 (0.01-1.62, p=0.205) |
| Sex | Male | 645 (89.3) | 77 (10.7) | - |
|  | Female | 76 (88.4) | 10 (11.6) | 1.25 (0.57-2.51, p=0.551) |
| BMI class | Underweight | 10 (90.9) | 1 (9.1) | - |
|  | Normal | 318 (90.3) | 34 (9.7) | 0.99 (0.17-18.88, p=0.991) |
|  | Overweight | 299 (86.9) | 45 (13.1) | 1.31 (0.23-24.96, p=0.804) |
|  | Obese | 75 (93.8) | 5 (6.2) | 0.61 (0.08-12.71, p=0.676) |
| ASA Physical Status | ASA 1-2 | 606 (89.0) | 75 (11.0) | - |
|  | ASA 3-4 | 114 (90.5) | 12 (9.5) | 0.92 (0.44-1.79, p=0.819) |
| Prostatectomy | No | 694 (89.4) | 82 (10.6) | - |
|  | Yes | 25 (83.3) | 5 (16.7) | 1.87 (0.59-5.02, p=0.242) |
| Previous hernia | No | 564 (89.4) | 67 (10.6) | - |
|  | Yes | 158 (88.8) | 20 (11.2) | 0.98 (0.54-1.70, p=0.953) |
| Operative technique | Open without mesh | 7 (87.5) | 1 (12.5) | - |
|  | Open with mesh | 684 (89.6) | 79 (10.4) | 0.88 (0.13-17.49, p=0.912) |
|  | Laparo-endoscopic with mesh | 31 (81.6) | 7 (18.4) | 1.83 (0.22-39.43, p=0.615) |
| Anaesthesia Modality | General | 444 (88.4) | 58 (11.6) | - |
|  | Regional-Local | 277 (90.8) | 28 (9.2) | 0.81 (0.48-1.33, p=0.408) |
| Hernia type | Femoral | 14 (93.3) | 1 (6.7) | - |
|  | Lateral | 360 (89.6) | 42 (10.4) | 1.47 (0.24-28.84, p=0.731) |
|  | Medial | 272 (88.0) | 37 (12.0) | 1.87 (0.30-36.97, p=0.574) |
|  | Mixed | 71 (92.2) | 6 (7.8) | 1.06 (0.13-22.95, p=0.964) |

**Supplementary Figure 2 - Multivariable logistic analysis of post-operative complication rate by mode of care**

| **Patient characteristics** |  | **No Complications** | **Complications** | **Multivariate OR** |
| --- | --- | --- | --- | --- |
| Setting | Day-case | 329 (89.6) | 38 (10.4) | - |
|  | Overnight stay | 247 (87.3) | 36 (12.7) | 1.11 (0.65-1.89, p=0.694) |
| Age (years) | 18-40 years | 64 (92.8) | 5 (7.2) | - |
|  | 41-60 years | 282 (89.8) | 32 (10.2) | 1.38 (0.55-4.20, p=0.530) |
|  | 61-80 years | 230 (86.1) | 37 (13.9) | 1.79 (0.70-5.54, p=0.257) |
|  | >80 years | 0 | 0 | - |
| Sex | Male | 512 (88.4) | 67 (11.6) | - |
|  | Female | 64 (90.1) | 7 (9.9) | 0.91 (0.36-2.03, p=0.838) |
| BMI | Normal | 261 (89.7) | 30 (10.3) | - |
|  | Overweight | 234 (86.3) | 37 (13.7) | 1.31 (0.78-2.24, p=0.309) |
|  | Obese | 63 (92.6) | 5 (7.4) | 0.71 (0.23-1.80, p=0.509) |
| Prostatectomy | No | 554 (88.6) | 71 (11.4) | - |
|  | Yes | 20 (87.0) | 3 (13.0) | 1.10 (0.25-3.56, p=0.880) |
| Previous hernia | No | 457 (88.6) | 59 (11.4) | - |
|  | Yes | 119 (88.8) | 15 (11.2) | 0.86 (0.43-1.60, p=0.649) |
| Operative technique | Open without mesh | 6 (85.7) | 1 (14.3) | - |
|  | Open with mesh | 545 (89.1) | 67 (10.9) | 0.71 (0.10-14.13, p=0.761) |
|  | Laparoendoscopic with mesh | 25 (80.6) | 6 (19.4) | 1.47 (0.17-32.19, p=0.752) |
| Anaesthesia General/Other | General | 371 (88.1) | 50 (11.9) | - |
|  | Regional-Local | 204 (89.9) | 23 (10.1) | 0.82 (0.46-1.42, p=0.491) |
| Hernia type | Femoral | 12 (92.3) | 1 (7.7) | - |
|  | Lateral | 297 (89.5) | 35 (10.5) | 1.19 (0.19-23.32, p=0.878) |
|  | Medial | 217 (86.8) | 33 (13.2) | 1.65 (0.27-32.41, p=0.655) |
|  | Mixed | 45 (91.8) | 4 (8.2) | 0.72 (0.07-16.51, p=0.796) |

**Supplementary Figure 3 - Multivariable logistic analysis of complication rate by mode of care in selected low-risk patients**
